# Inoculating for Cholera

**Published:** 1885-08

**Authors:** 


### Inoculating for Cholera.

*Science,* in an article giving the details of the inoculation experiments made in Spain, and reported to the French Academy, says : "The writer draws these conclusions from his experiments, and offers to reproduce his results before the Academy : 1st. 'Cholerization is possible in man, as in animals, by hypodermic injection. 2d. That the prophylaxis of cholerization is obtained through graduated doses or attenuated virus.'" *Science* adds, for its own opinion, "These experiments are said to have been carried on further, but no proper report of them has yet reached us. Our criticism would be that the conclusion as to the efficacy of inoculation against cholera, granting thet the true bacillus of cholera was used, is an exceedingly hasty one, inasmuch as the protected (?) persons have not yet been brought in contact with the disease."

